# Simulation-based approach with an adaptive Type-II hybrid progressive censoring scheme to study the coefficient of variation of the weighted exponential distribution

**DOI:** 10.1371/journal.pone.0337982

**Published:** 2026-08-27

**Authors:** Hassan M. Aljohani, Amirah Saeed Alharthi, Wafa Ali J. Almohri, Sadiah M. A. Aljeddani, Nahaa Eid Alsubaie, Walaa A. El-Sharkawy

**Affiliations:** 1 Department of Mathematics and Statistics, College of Science, Taif University, Taif, Saudi Arabia; 2 Mathematics Department, Faculty of Science, Taibah University, Al-Madinah Al-Munawarah, Kingdom of Saudi Arabia; 3 Mathematics Department, Al-Lith University College, Umm Al-Qura University, Al-Lith, Saudi Arabia; 4 Department of Mathematics and Statistics, University College of Khurma, Taif University, Taif, Saudi Arabia; 5 Department of Mathematics, Faculty of Science, Cairo University, Giza, Egypt; Memorial Sloan Kettering Cancer Center, UNITED STATES OF AMERICA

## Abstract

The coefficient of variation (CV) is a frequently used standardized measure of variability that allows meaningful comparison among datasets measured using different scales or units. Although widely applied in practice, formal statistical inference for the CV under non-normal distributions has received limited attention. This study focuses on estimating the CV for the weighted exponential distribution under an adaptive Type-II hybrid progressive censoring scheme, which enhances the efficiency of life-testing experiments by balancing test duration and the number of observed failures. Both Bayesian and non-Bayesian frameworks are considered. Point estimation in the non-Bayesian framework is carried out using the maximum likelihood method, whereas interval estimates are constructed through the parametric bootstrap approach. For the Bayesian approach, posterior inference is performed through Markov chain Monte Carlo sampling with under appropriate gamma prior assumptions. To illustrate the implementation of the proposed procedures, a simulated dataset is analyzed, and a comprehensive Monte Carlo simulation study is subsequently conducted to evaluate the accuracy and efficiency of the estimators. According to the analysis, Bayesian estimators based on informative prior distributions yield more precise estimates of the CV.

## 1. Introduction

The coefficient of variation (CV) is a standard statistical metric defined as the ratio of the population standard deviation to the population mean. It is used to evaluate relative variability or uncertainty of data and is particularly useful when comparing datasets measured in different units. By standardizing the measure of dispersion, the CV allows for meaningful comparisons across diverse datasets, such as comparing weights in grams and heights in centimeters.

CV has been extensively utilized across both theoretical developments and practical applications in statistics. Pang et al. [1] proposed a simulation-based Bayesian approach for estimating the population CV under the three-parameter gamma distribution, with extensions to the lognormal and Weibull models. This methodology was later adapted by Pang et al. [2] to the beta distribution for estimating the CV of bounded data, such as dividend yields in the Hong Kong stock market. Further contributions include CV estimation for Gompertz distribution by Soliman et al. [3] and for compound Rayleigh populations by Al-Aboud et al. [4]. Beyond theoretical modeling, the CV is widely applied in clinical and diagnostic settings to assess measurement accuracy, as shown by Reh and Scheffler [5]. It is also used in physiological and biomedical research, for instance, to evaluate the homogeneity of bone samples as demonstrated by Hamer et al. [6] and in finance to quantify relative risk as discussed by Miller and Karson [7].

Although the CV is widely used to measure variability, its estimation often relies on complete data. However, in life testing experiments, collecting complete data is often impractical due to time constraints, costs, or other constraints. Consequently, researchers often work with censored data, where only partial information is available. To accommodate such settings, various censoring schemes (CSs), such as Type-I, Type-II, progressive, and hybrid, have been developed to enable efficient analysis under incomplete data conditions.

Among these, the most common are Type-I and Type-II CSs. In a Type-I CS, the experiment is terminated at a predetermined time point η, with the number of observed failures being random. In contrast, a Type-II CS ends the experiment once a fixed number of failures *m* has occurred, making the test duration random. While both approaches are practical, they offer limited flexibility, as units can only be removed from the test at its termination.

This lack of adaptability motivated the development of a more general censoring framework, known as the progressive censoring scheme (PCS). Under this scheme, greater adaptability is achieved by allowing the removal of units at intermediate stages of the experiment; see Balakrishnan and Aggarwala [8] for more details. In a Type-II PCS, the experiment begins with *n* independent and identically distributed units drawn from the population, and both the number of failures *m* and the CS $𝐫=(r_{1},r_{2},...,r_{m})$ are specified in advance. At each observed failure time $T_{i;m,n}^{𝐫}$, $r_{i}$ surviving units are randomly withdrawn, for i=1,2,...,m, yielding a progressively censored sample ${𝐓}^{𝐫}=(T_{1;m,n}^{𝐫},T_{2;m,n}^{𝐫},...,T_{m;m,n}^{𝐫})$.

The hybrid censoring scheme (HCS) represents an extension of traditional CSs by incorporating both Type-I and Type-II features. Specifically, the Type-I hybrid progressive censoring scheme (Type-I HPCS) allows the experiment to terminate at the earlier of a fixed time η or the *m*-th failure time, while still enabling the removal of units at each failure point. Under this CS, at each failure time $T_{i;m,n}^{𝐫}$, $r_{i}$ surviving units are withdrawn from the test for i=1,2,...,k. The experiment continues until the earlier occurrence of the predetermined time η and the *m*-th failure time, i.e., min$\left(\eta ,T_{m;m,n}^{𝐫}\right)$. The resulting failure times ${𝐓}^{𝐫}=(T_{1;m,n}^{𝐫},T_{2;m,n}^{𝐫},...,T_{k;m,n}^{𝐫}),$ where k≤m, constitute a Type-I HPCS sample. As η→∞, this scheme reduces to the standard Type-II PCS. For more details on HCSs and their properties, see Kundu and Joarder [9] and Abushal et al. [10].

Although Type-II PCS and Type-I HPCS are more flexible than traditional censoring methods, they still have an important drawback. In both cases, either the number of observed failures or the total test duration remains random. This can result in insufficient failure data or unnecessarily long experiments. To address this, the adaptive Type-II hybrid progressive censoring scheme (adaptive Type-II HPCS) was introduced; see Ng et al. [11], Abd-Elmougod and Mohamed [12], and Abd-Elmougod et al. [13]. This scheme specifies both a target number of failures *m* and a planned termination time η, allowing the experiment to stop at $T_{m;m:n}$ if the *m*-th failure occurs before η, or to terminate as soon as possible after η if fewer than *m* failures are observed.

Under this framework, if the planned termination time η satisfies


$$\begin{array}{c} T_{J;m:n}<\eta <T_{J+1;m:n},\text{ for some }J=1,2,...,m, \end{array}$$

with $T_{0;m:n}=0\text{ and }T_{m+1;m:n}=\infty$, then the remaining censoring values $r_{J+1},r_{J+2},...,r_{m-1}$are set to zero. The final censoring value $r_{m}$ is modified as $r_{m}=n-m-\sum_{i=1}^{J}r_{i}$. This adjustment ensures a higher probability of capturing extreme failures while reducing the total test time. For a detailed statistical foundation of this approach, see David and Nagaraja [14] and Ng and Chan [15].

**Fig 1:**
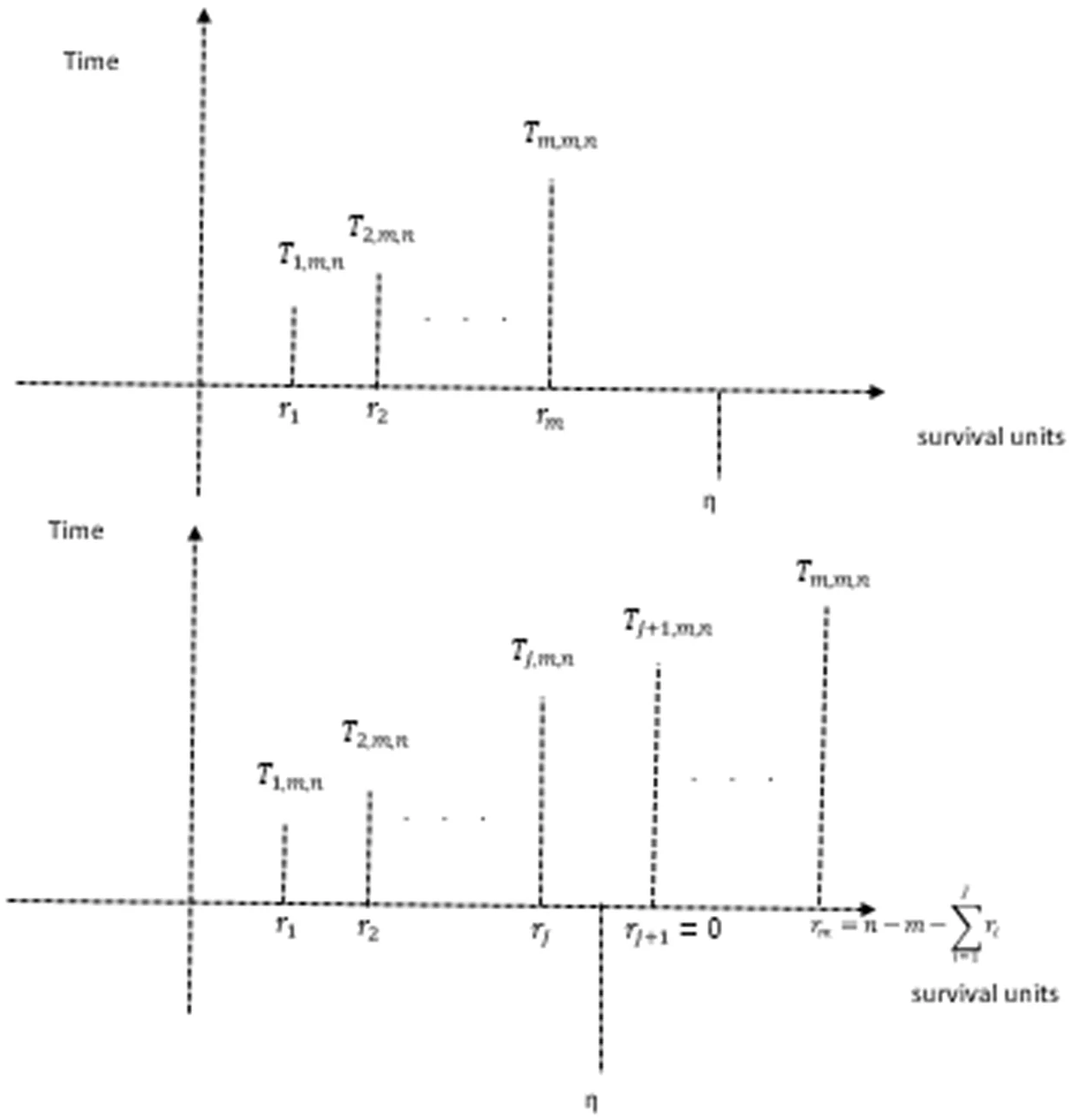
Adaptive Type-II HPCS when $T_{m;m:n}\leq \eta$ and $T_{m;m:n}>\eta$.

The ordinary Type-II PCS is recovered as a special case of an adaptive Type-II HPCS when η→∞, while the standard Type-II CS corresponds to the case when η=0. Consequently, an adaptive Type-II HPCS enables more effective control over both test duration and the number of observed failures, resulting in more reliable and efficient statistical inference. This flexibility is illustrated in Fig 1.

Under an adaptive Type-II HPCS, let $T=\{T_{1;m:n},T_{2;m:n},...,T_{J;m:n},T_{J+1;m:n},...,T_{m;m:n}\}$ denote the observed progressively censored sample. The associated likelihood function is expressed as


$$\begin{array}{cc} f_{1,2,...,m}(t_{1},t_{2},...,t_{m}|\theta )=\; & Q\prod_{i=1}^{m}f(t_{i})[1-F(t_{i}){]}^{r_{i}^{*}},\\ & 0<t_{1}<t_{2}<...<t_{m}<\infty , \end{array}$$ (1)

where the multiplicative constant *Q* is given by


$$\begin{array}{c} Q=\prod_{i=1}^{m}\left[n-i+1-\sum_{i=1}^{\min\left\{i-1,J\right\}}r_{i}\right]. \end{array}$$ (2)

Here, the adjusted censoring values $r_{i}^{*}$ are defined as


$$\begin{array}{c} r_{i}^{*}=\left\{ \begin{array}{cc} r_{i}, & \text{if}\;i\leq J,\\ 0, & \text{if}\;J<i<m,\\ n-m-\sum_{j=1}^{J}r_{j}, & \text{if}\;i=m\text{ and }J<m. \end{array}\right. \end{array}$$ (3)

This formulation allows for modeling different censoring scenarios depending on how many failures occur before the ideal test time η, making an adaptive Type-II HPCS highly versatile.

In addition to CSs, the choice of an appropriate lifetime distribution plays a vital role in achieving accurate statistical modeling. Among these distributions, the weighted exponential (WE) distribution, originally developed by Gupta and Kundu [16], enhances the standard exponential distribution by incorporating a shape parameter *a* > 0 alongside a scale parameter *b* > 0. This extension provides increased flexibility for modeling various types of survival data. The WE distribution has been shown to perform better than other common distributions like the Weibull, gamma, and generalized exponential, especially when the failure rate is not constant. Let *T* be a random variable following the WE distribution with parmeters *a* and *b*, denoted by T~WE(a,b). Its probability density function (PDF) is defined as


$$\begin{array}{c} f(t)=\frac{a+1}{a}be^{-bt}(1-e^{-abt}),\;t>0. \end{array}$$ (4)

The distribution approaches the standard exponential model with parameter *b* as a→∞, and to gamma distribution Γ(2, *b*) as a→0. Furthermore, the distribution is unimodal, with its mode located at log(1+a)/a.

The cumulative distribution function (CDF) and hazard rate function (HRF) are given, respectively, by


$$\begin{array}{c} F(t)=1-\frac{1}{a}e^{-bt}(a+1-e^{-abt}), \end{array}$$ (5)

and


$$\begin{array}{c} H(t)=(a+1)b\frac{1-e^{-abt}}{a+1-e^{-abt}}. \end{array}$$ (6)

**Fig 2:**
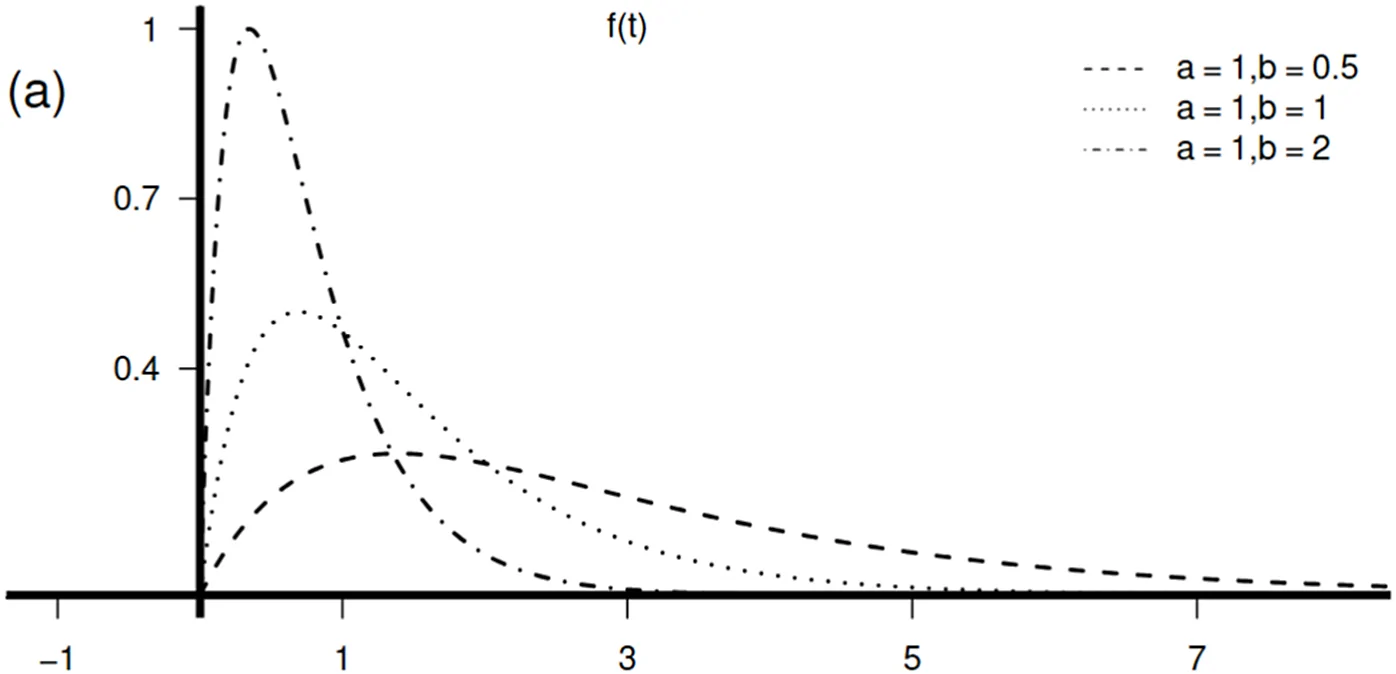
Plots of PDF of the WE distribution for selected parameter values.

**Fig 3:**
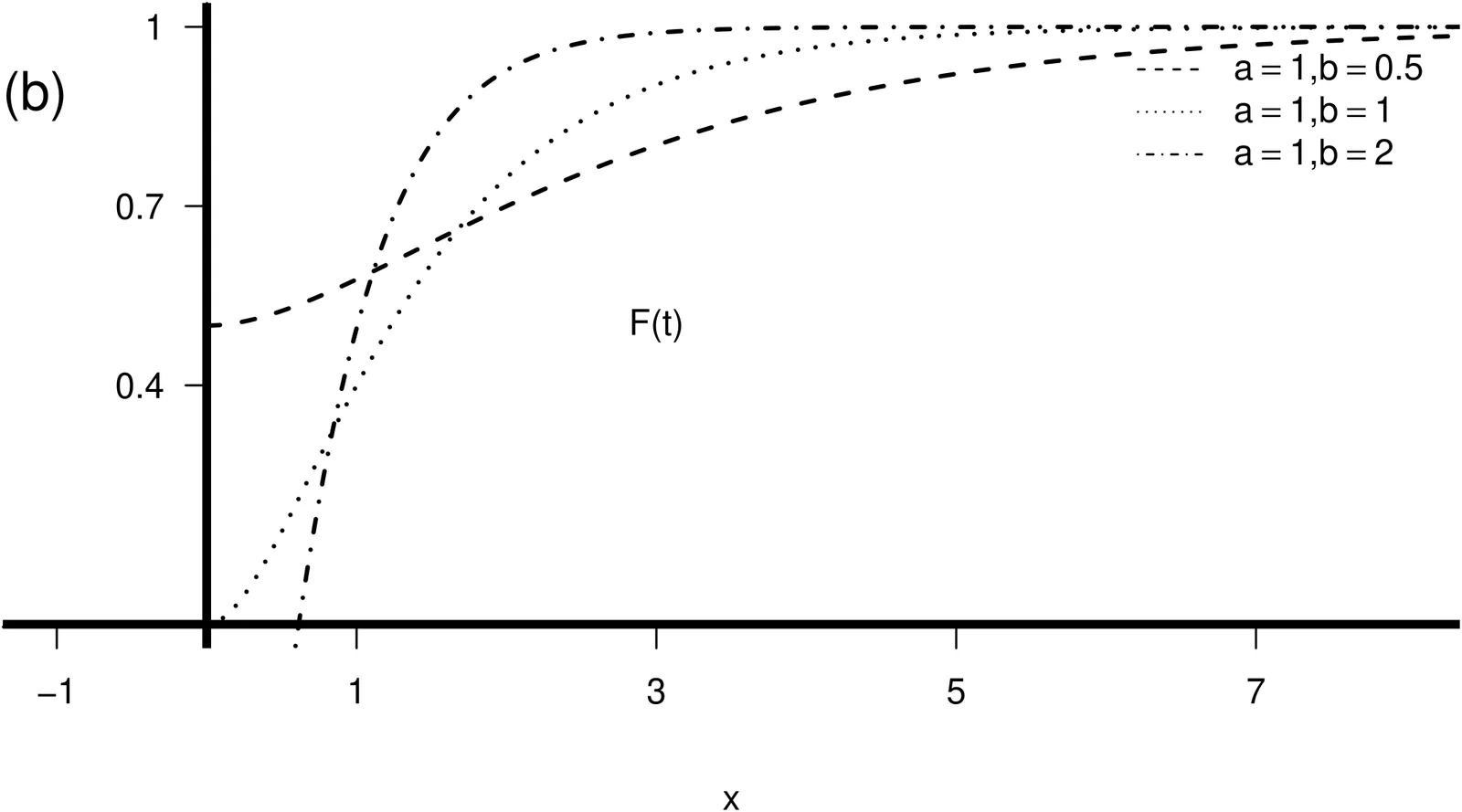
Plots of CDF of the WE distribution for selected parameter values.

**Fig 4:**
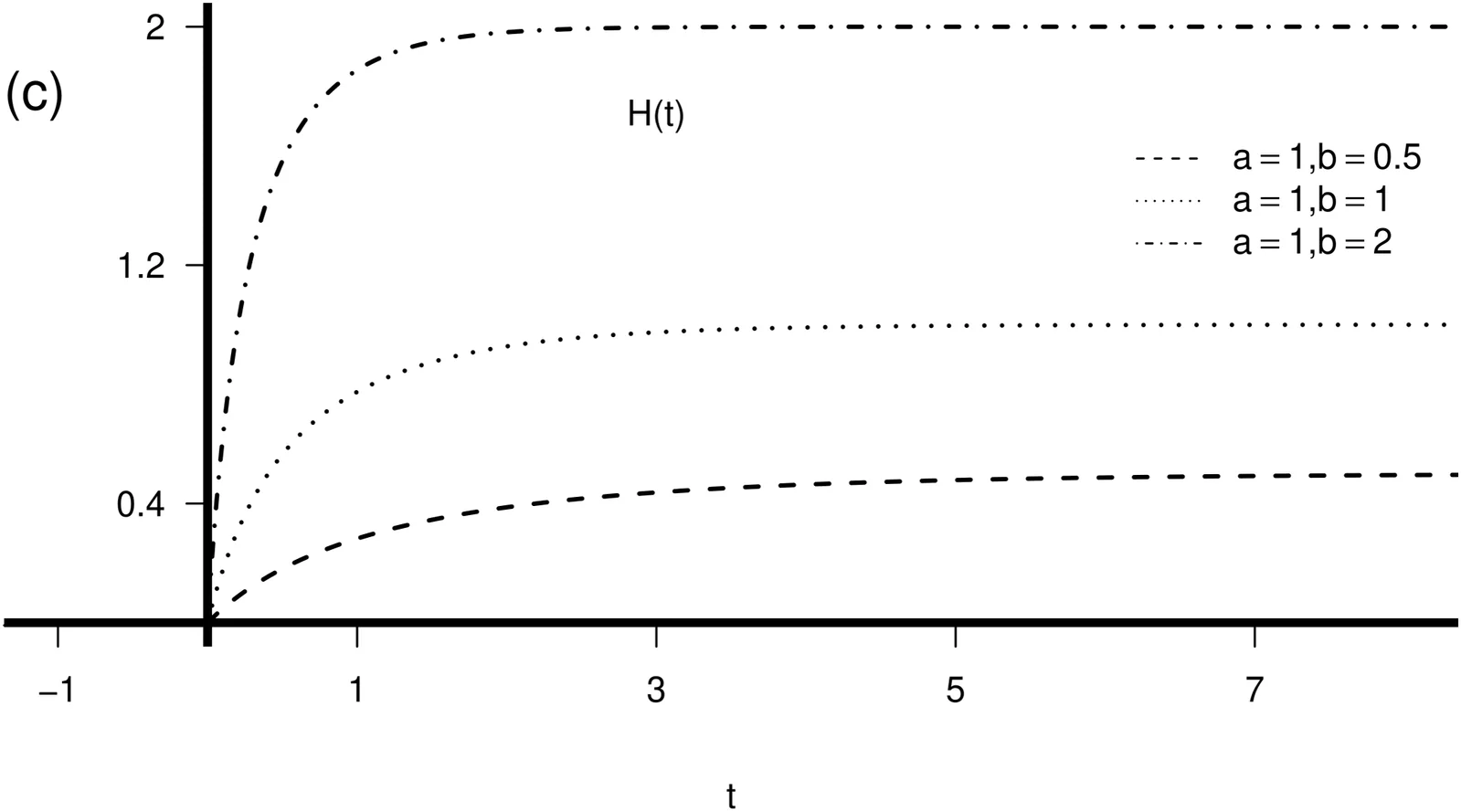
Plots of HRF of the WE distribution for selected parameter values.

Several studies have explored the estimation methods for the WE distribution. In particular, Alqallaf et al. [17] investigated different parameter estimation techniques, while Shakhatreh [18] introduced a broader family of two-parameter WE distribution. Figs 2–4 illustrate how the WE distribution responds to changes in the scale parameter *b*, with the shape parameter fixed at *a* = 1. In Fig 2, the PDF plots become more peaked and shift to the left as *b* increases, indicating shorter lifetimes, while smaller *b* values produce heavier tails, suggesting longer survival times. Fig 3 shows that the CDF plots rise more quickly for larger *b*, reflecting faster failure accumulation. Fig 4 presents the HRF curves, which increase over time for all values of *b*; larger *b* values result in steeper hazard growth. These visualizations emphasize the flexibility of the WE distribution in capturing lifetime data with increasing failure rates.

The WE distribution exhibits several useful properties. Specifically, its first two raw moments are given, respectively, by


$$\begin{array}{c} \mu_{1}=\frac{\left(a+2\right)}{b\left(a+1\right)}, \end{array}$$ (7)

and


$$\begin{array}{c} \mu_{2}=\frac{2(a^{2}+3a+2)}{b^{2}{\left(a+1\right)}^{2}}. \end{array}$$ (8)

Based on these moments, the theoretical CV can be expressed as


$$\begin{array}{c} \text{CV}=\frac{\sqrt{\mu_{2}-\mu_{1}^{2}}}{\mu_{1}}=\sqrt{\frac{a}{a+2}}. \end{array}$$ (9)

The primary objective of this study is to develop and assess statistical procedures for estimating the CV of the WE distribution within the framework of the adaptive Type-II HPCS. The WE distribution is selected for its ability to model lifetime data with non-constant hazard rates, while the adaptive CS improves efficiency by optimizing the trade-off between the number of observed failures and the total test duration. Estimation is performed under both non-Bayesian and Bayesian methods. In the non-Bayesian approach, maximum likelihood (ML) estimation is applied to estimate the CV, and confidence intervals are derived using the bootstrap percentile method. For Bayesian analysis, Markov chain Monte Carlo (MCMC) methods are employed to compute the posterior estimates. The effectiveness of the proposed methods is examined through simulation studies and illustrated using a simulated dataset.

The paper is arranged in the following manner. Section 2 describes the estimation of the model parameters and the CV using both ML and Bayesian approaches. Section 3 focuses on constructing interval estimates for the CV, including parametric percentile bootstrap confidence intervals and Bayesian credible intervals. Section 4 provides an illustrative analysis based on a simulated dataset, while Section 5 presents a Monte Carlo simulation study to evaluate the performance of the CV estimators under various scenarios. Final conclusions are summarized in Section 6.

## 2. Point Estimation

This section addresses the estimation of the WE distribution parameters *a*, *b*, and CV under an adaptive Type-II HPCS. The analysis includes both the ML approach and Bayesian inference using MCMC techniques.

### 2.1 Maximum Likelihood Estimation

Let $𝐓=\{T_{1;m:n},T_{2;m:n},...,T_{J;m:n},T_{J+1;m:n},...,T_{m;m:n}\}$ denote the observed failure times under an adaptive Type-II HPCS. Given that the lifetimes follow the WE distribution with PDF and CDF given in Eqs (4) and (5), respectively, the joint likelihood function corresponding to this scheme, as expressed in Eq (1), can be written as follows


$$\begin{array}{cc} L(a,b|𝐭)=\; & Q{\left(\frac{1}{a}\right)}^{n}{\left(a+1\right)}^{m}(b{)}^{m}\exp\{-b\sum_{i=1}^{m}(r_{i}^{*}+1)t_{i}+\sum_{i=1}^{m}\log[1-e^{-abt_{i}}]\\ & +\sum_{i=1}^{m}r_{i}^{*}\log[a+1-e^{-abt_{i}}]\}, \end{array}$$ (10)

where *Q* is given by Eq (2). The logarithm of the likelihood function in Eq (10), excluding the normalizing constant *Q*, is given by


$$\begin{array}{ccc} \ell (a,b|𝐭) & = & -n\log a+m\log\left[a+1\right]+m\log b-b\sum_{i=1}^{m}(r_{i}^{*}+1)t_{i}\\ & & +\sum_{i=1}^{m}\log\left[1-e^{-abt_{i}}\right]+\sum_{i=1}^{m}r_{i}^{*}\log[a+1-e^{-abt_{i}}]. \end{array}$$ (11)

By taking the first partial derivatives of Eq (11), the likelihood equations for the parameters *a* and *b*, respectively, are obtained as follows


$$\begin{array}{c} \frac{\partial \ell \left(a,b|𝐭\right)}{\partial a}=\frac{-n}{a}+\frac{m}{a+1}+b\sum_{i=1}^{m}\frac{t_{i}e^{-abt_{i}}}{1-e^{-abt_{i}}}+\sum_{i=1}^{m}\frac{r_{i}^{*}\left(1+bt_{i}e^{-abt_{i}}\right)}{a+1-e^{-abt_{i}}}=0, \end{array}$$ (12)

and


$$\begin{array}{c} \frac{\partial \ell \left(a,b|𝐭\right)}{\partial b}=\frac{m}{b}-\sum_{i=1}^{m}(r_{i}^{*}+1)t_{i}+a\sum_{i=1}^{m}\frac{t_{i}e^{-abt_{i}}}{1-e^{-abt_{i}}}+a\sum_{i=1}^{m}\frac{r_{i}^{*}t_{i}e^{-abt_{i}}}{a+1-e^{-abt_{i}}}=0. \end{array}$$ (13)

These two non-linear equations in *a* and *b* can be solved numerically using the Newton-Raphson method to obtain the ML estimates $\hat{a}$ and $\hat{b}$. Therefore, the ML estimate of the CV is


$$\begin{array}{c} \hat{\text{CV}}=\sqrt{\frac{\hat{a}}{\hat{a}+2}}. \end{array}$$ (14)

### 2.2. Bayesian estimation

Bayesian approaches commonly utilize gamma prior distributions for modeling positive valued parameters. For the WE distribution, this choice is particularly suitable for the parameters *a* and *b*, as the gamma distribution is defined on the positive real line, which is consistent with the domain of these parameters. It also takes on a variety of shapes, from exponential to approximately normal distributions, depending on the choice of hyperparameters, reflecting its flexibility. Assuming independent gamma priors for the unknown parameters *a* and *b*, the joint prior distribution is given by


$$\begin{array}{c} \pi^{*}(a,b)=\pi_{1}^{*}(a|\beta_{1},\theta_{1})\pi_{2}^{*}(b|\beta_{2},\theta_{2}),\;\beta_{1},\theta_{1},\beta_{2},\theta_{2}>0;\;a,b>0, \end{array}$$ (15)

where


$$\begin{array}{c} \pi_{1}^{*}(a|\beta_{1},\theta_{1})\propto a^{\beta_{1}-1}\exp\left[-\theta_{1}a\right], \end{array}$$ (16)

and


$$\begin{array}{c} \pi_{2}^{*}(b|\beta_{2},\theta_{2})\propto b^{\beta_{2}-1}\exp\left[-\theta_{2}b\right]. \end{array}$$ (17)

From the joint likelihood function in Eq (10) and the joint prior distribution in Eq (15), the joint posterior density of the parameters *a* and *b* is given by


$$\begin{array}{cc} \pi (a,b|t) & \propto a^{-n+\beta_{1}-1}(a+1{)}^{m}b^{m+\beta_{2}-1}\exp\{-\theta_{1}a-\theta_{2}b-b\sum_{i=1}^{m}(r_{i}^{*}+1)t_{i}\\ & +\sum_{i=1}^{m}\log[1-e^{-abt_{i}}]+\sum_{i=1}^{m}r_{i}^{*}\log[a+1-e^{-abt_{i}}]\}. \end{array}$$ (18)

Assuming a squared error loss function, the Bayes estimator of an arbitrary function ϕ(a,b), including *a*, *b*, and CV, is given by


$$\begin{array}{c} \hat{\phi }(a,b{)}_{\text{B}}=\frac{\int_{0}^{\infty }\int_{0}^{\infty }\phi (a,b)L\left(a,b|𝐭\right)\times \pi^{*}(a,b)dadb}{\int_{0}^{\infty }\int_{0}^{\infty }L\left(a,b|𝐭\right)\times \pi^{*}(a,b)dadb}. \end{array}$$ (19)

Evaluating the posterior distribution in Eq (18) and the corresponding Bayes estimate in Eq (19) involves computing a high-dimensional integral, which makes direct calculation challenging. Due to the analytical complexity of the posterior distribution, MCMC approach is employed to evaluate the posterior distribution and determine the associated Bayes estimates, as detailed below.

#### 2.2.1. MCMC approach.

We consider the MCMC method to generate samples from the posterior distributions and compute the Bayes estimates of the CV for the WE distribution using the squared error loss function. While numerous MCMC schemes are available, selecting an appropriate approach can be challenging. Among the most widely used and effective techniques are Gibbs sampling and the more general Metropolis-within-Gibbs algorithm. The joint posterior distribution given in Eq (18) can be decomposed into the following full conditional distributions:


$$\begin{array}{c} \pi_{1}(a|b,t)\propto a^{-n+\beta_{1}-1}{\left(a+1\right)}^{m}\exp\left\{-\theta_{1}a+\sum_{i=1}^{m}\log\left[1-e^{-abt_{i}}\right]+\sum_{i=1}^{m}r_{i}^{*}\log\left[a+1-e^{-abt_{i}}\right]\right\}, \end{array}$$ (20)

and


$$\begin{array}{cc} \pi_{2}(b|a,t) & \propto b^{m+\beta_{2}-1}\exp\{-b\theta_{2}+\sum_{i=1}^{m}(r_{i}^{*}+1)t_{i}+\sum_{i=1}^{m}\log[1-e^{-abt_{i}}]\\ & +\sum_{i=1}^{m}r_{i}^{*}\log[a+1-e^{-abt_{i}}]\}. \end{array}$$ (21)

Since the posterior reduces to conditional distributions of *a* and *b*, we apply the Metropolis-Hastings algorithm within a Gibbs sampler using normal proposals. This approach efficiently generates posterior samples for estimating the Bayes estimator of ϕ(a,b). The algorithm steps are described below.

**Remark:** Under the MCMC method, the convergence of the chain is visually assessed using the trace plot and histogram. When the trace plot shows stable fluctuations around a constant mean without noticeable trends, this indicates good mixing and convergence of the chain. Also, the stability of the MCMC estimates is indicated by the histogram, which displays a unimodal and approximately normal posterior distribution.

**Algorithm 1 Metropolis-within-Gibbs algorithm**

1: **Input:** Conditional distributions (20) and (21) with objective proposal function, bivariate normal distribution.

2: **Output:** Empirical posterior distribution of ϕ=ϕ(a,b).

3: Begin with initial values $a^{\left(0\right)}=\hat{a}$ and $b^{\left(0\right)}=\hat{b}\;$and put κ=1.

4: Generate $a^{\left(\kappa \right)}$ from (20) and $b^{\left(\kappa \right)}$ from (21) using the Metropolis-Hastings algorithm with normal proposals with mean ($\hat{a}$, $\hat{b}$) and variance taken from approximate information matrix.

5: Compute $\phi^{\left(\kappa \right)}$ from (9).

6: Set κ=κ+1.

7: Repeat steps 2–4 *N* times to obtain $\phi^{\left(1\right)},\phi^{\left(2\right)},...,\phi^{\left(N\right)}$.

Based on the *N* samples generated using Algorithm 1, the initial *M* samples are excluded as burn-in to eliminate the influence of initial values. The Bayes estimator of the ϕ under the squared error loss function is then computed as the posterior mean of the remaining (N−M) samples:


$$\begin{array}{c} {\hat{\phi }}_{\text{B}}=\frac{1}{N-M}\sum_{i=M+1}^{N}\phi^{\left(i\right)}, \end{array}$$ (22)

where *M* is the burn-in period.

## 3. Interval estimation

This section presents methods for constructing interval estimates for the CV of the WE distribution under an adaptive Type-II HPCS. Both parametric bootstrap intervals and MCMC-based Bayesian credible intervals are investigated.

### 3.1. Bootstrap confidence intervals

Confidence interval estimation using bootstrap techniques has been extensively investigated in the literature; see Davison and Hinkley [19] and Efron and Tibshirani [20] for comprehensive discussions. The parametric percentile bootstrap method frequently yields confidence intervals with empirical coverage probabilities that are close to the nominal confidence level. In this subsection, we use the percentile bootstrap method to construct confidence intervals for the CV of the WE distribution under an adaptive Type-II HPCS, following approaches from Efron [21], Hall [22], and Abed-Elmougod et al. [13]. The procedure for constructing the bootstrap confidence intervals is summarized in the following algorithm:

**Algorithm 2 Bootstrap confidence intervals of CV**

1: **Input:** Object function (5), ML estimates $\hat{a}$ and $\hat{b}$, scheme parameter {n,m,η,𝐫} and the bootstrap iteration size *N* = 500.

2: **Output:** Bootstrap sample estimate of CV

3: Generate a bootstrap sample ${𝐓}^{*}=\{T_{1;m:n}^{*},T_{2;m:n}^{*},...,T_{m;m:n}^{*}\}$ from the WE distribution using $\hat{a}$ and $\hat{b}$, as described in Ng et al. [11].

4: For each bootstrap sample, compute new estimates ${\hat{a}}^{*},{\hat{b}}^{*}$, and the corresponding ${\hat{CV}}^{*}$.

5: Steps 3 and 4 are repeated *N* times, getting *N* bootstrap estimates of CV based on *N* different bootstrap samples, denoted ${\hat{\text{CV}}}_{j}^{*},\text{ for }j=1,2,...,N$.

6: Arrange the bootstrap estimates $\hat{{\text{CV}}_{j}^{*}}$ in ascending order to obtain the ordered sequence: $\left({\hat{\text{CV}}}_{\left(1\right)}^{*},{\hat{\text{CV}}}_{\left(2\right)}^{*},...,{\hat{\text{CV}}}_{\left(N\right)}^{*}\right)$.

Defining $H(x)=P({\hat{\text{CV}}}^{*}⩽x)$ as the CDF of the bootstrap estimates ${\hat{\text{CV}}}^{*}$, the bootstrap quantile estimate for a given *x* is defined as ${\hat{\text{CV}}}_{\text{Boot}}^{*}=H^{-1}(x)$. Therefore, 100(1−2α)% approximate confidence interval of ${\hat{\text{CV}}}^{*}$ is given by


$$\begin{array}{c} \left[{\hat{\text{CV}}}_{\text{Boot(}\alpha \text{)}}^{*},{\hat{\text{CV}}}_{\text{Boot(1-}\alpha \text{)}}^{*}\right]. \end{array}$$ (23)

### 3.2. Bayesian credible intervals

To construct the Bayesian credible intervals for the CV, the posterior sample $\{{\hat{\text{CV}}}^{\left(i\right)}{\}}_{i=M+1}^{N}$, obtained from Eq (22) is used. This sample is then ordered, and the symmetric credible interval 100(1−2α)% is given by


$$\begin{array}{c} \left({\text{CV}}_{\left((N-M)\alpha \right)},{\text{CV}}_{\left((N-M)(1-\alpha )\right)}\right). \end{array}$$ (24)

## 4. Illustrative example

This section presents a simulated example based on data generated from the WE distribution to demonstrate the effectiveness of the proposed methods. The simulated dataset is generated using the algorithm described by Ng et al. [11] (Algorithm 3), and is used to illustrate the application of the techniques discussed earlier.

**Algorithm 3 Generate an adaptive Type-II HPC data**

1: **Input:** Suppose, the sample size *n* = 30, effected sample size *m* = 20, PCS *r*={1, 0, 1, 0, 1, 0, 1, 0, 1, 0, 1, 0, 1, 0, 1, 0, 1, 0, 1, 0}, η=2.0, distribution parameters *a* = 1.5 and *b* = 0.5 and objective function (5)

2: **Output:** An adaptive Type-II HPC data.

3: Generate *m* Type-II PC failure times $t_{1},t_{2},...,t_{m}$ from (5) using algorithms given in Balakrishnan and Sandhu [23].

4: Compare the data with time η to determine the integer value *J*.

5: If m≤J go to Step 7.

6: Generate the first (m−J) ordered data from left truncated distribution of (5) at $t_{J,m,n}$.

7: $t_{1},t_{2},...,t_{J},t_{J+1},...,t_{m}$.

8: **Return**, *J* and the adaptive Type-II HPC data.

Algorithm 3 yields a value of *J* = 11, with the corresponding simulate failure times are given by t={0.439865, 0.76812, 0.849096, 0.89154, 1.16056, 1.37031, 1.56963, 1.70367, 1.8577, 1.87114, 1.93648, 2.4105, 2.62075, 2.82089, 3.38811, 3.48332, 3.67979, 3.73976, 4.90813, 5.40977}.

In the Bayesian analysis, prior distributions were chosen such that the prior means satisfy ($E(a)=\frac{\beta_{1}}{\theta_{1}}≃a\text{ and }E(b)=\frac{\beta_{2}}{\theta_{2}}≃b$). The MCMC algorithm was run for 11,000 iterations, discarding the first 1,000 as burn-in.

All numerical computations were carried out using Mathematica (Version 10). The simulated data were generated according to Algorithm 3. ML estimates $\hat{a}$ and $\hat{b}$ were obtained using Eqs (12) and (13), respectively, while Eq (14) was used to compute $\hat{\text{CV}}$. For Bayesian point estimation, Algorithm 1 was implemented in conjunction with Eq (22). Interval estimation for the CV was performed according to Algorithm 2: bootstrap confidence intervals were constructed using Eq (23), and Bayesian credible intervals were obtained using Eq (24), with all intervals computed at the 95%. Similar procedures were applied to obtain the interval estimates for the parameters *a* and *b*. The resulting point and interval estimates are reported in Table 1.

**Table 1:** The point and interval estimates of MLE, Bootstrap, and MCMC for the parameters *a*, *b*, and CV.

Parameter	Exact	(.)_ML_	(.)_Bayes_	(95%)_Boot_	(95%)_MCMC_
a	1.5000	1.3990	1.4213	(0.4254, 2.8450)	(0.5842, 2.9214)
b	0.5000	0.8203	0.8203	(0.1452, 1.4213)	(0.2254, 1.5214)
CV	0.6124	0.6416	0.6241	(0.4215, 0.7214)	(0.4215, 0.7214)

**Fig 5:**
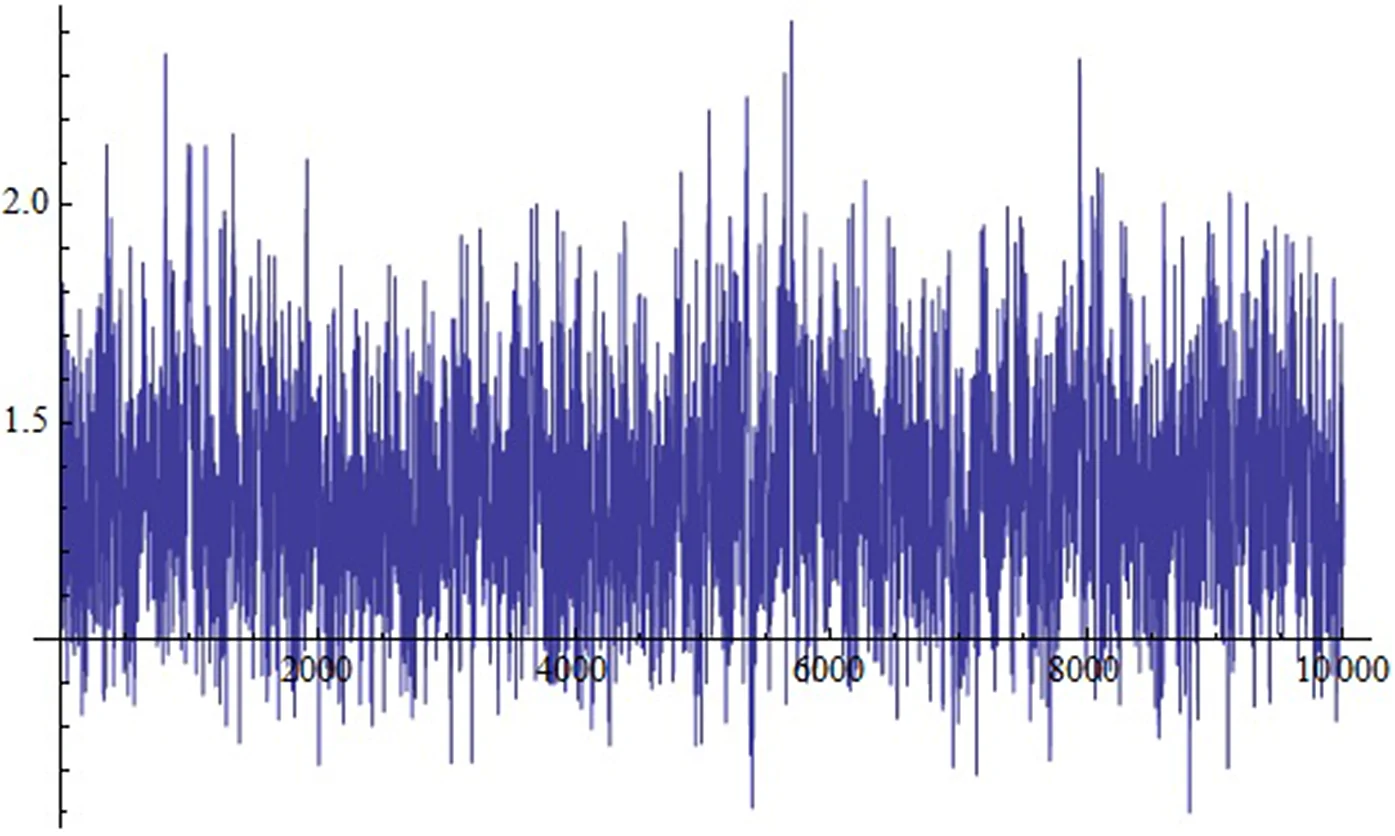
Simulation number of the parameter *a* generated by MCMC method.

**Fig 6:**
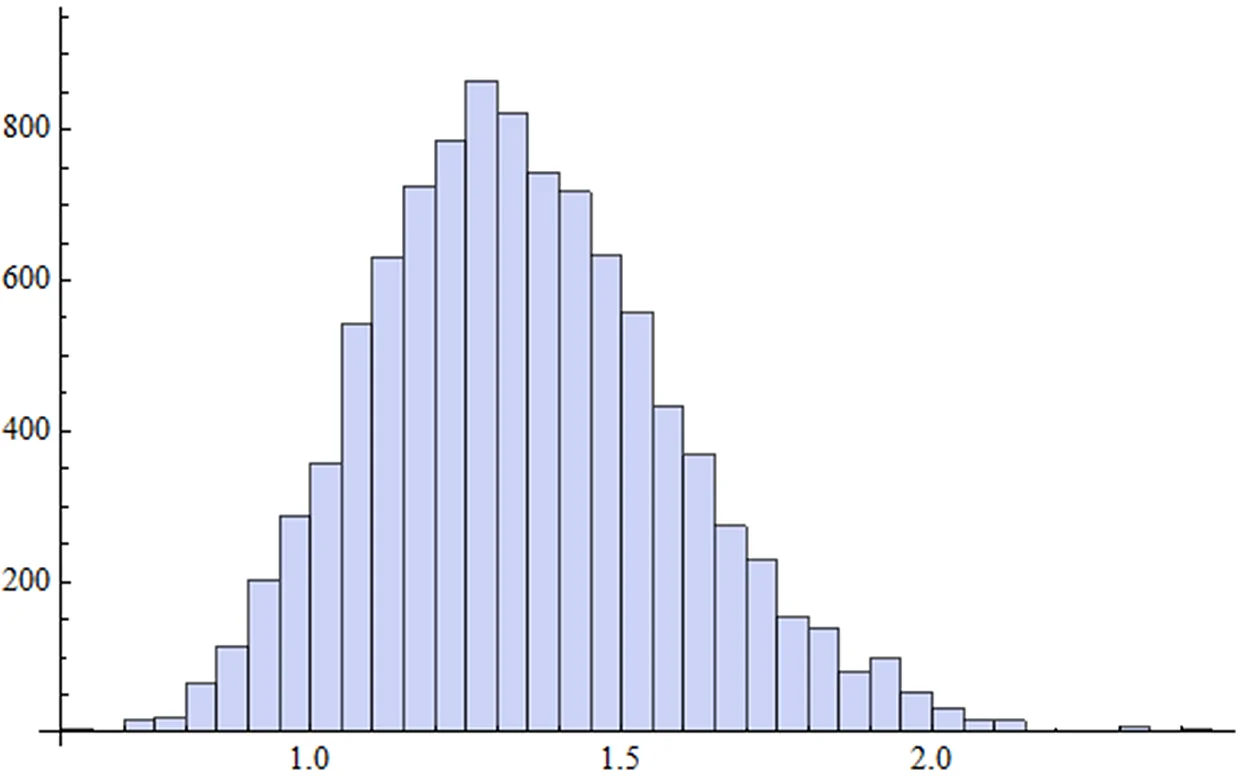
Histogram of posterior distribution of the parameter *a.*

**Fig 7:**
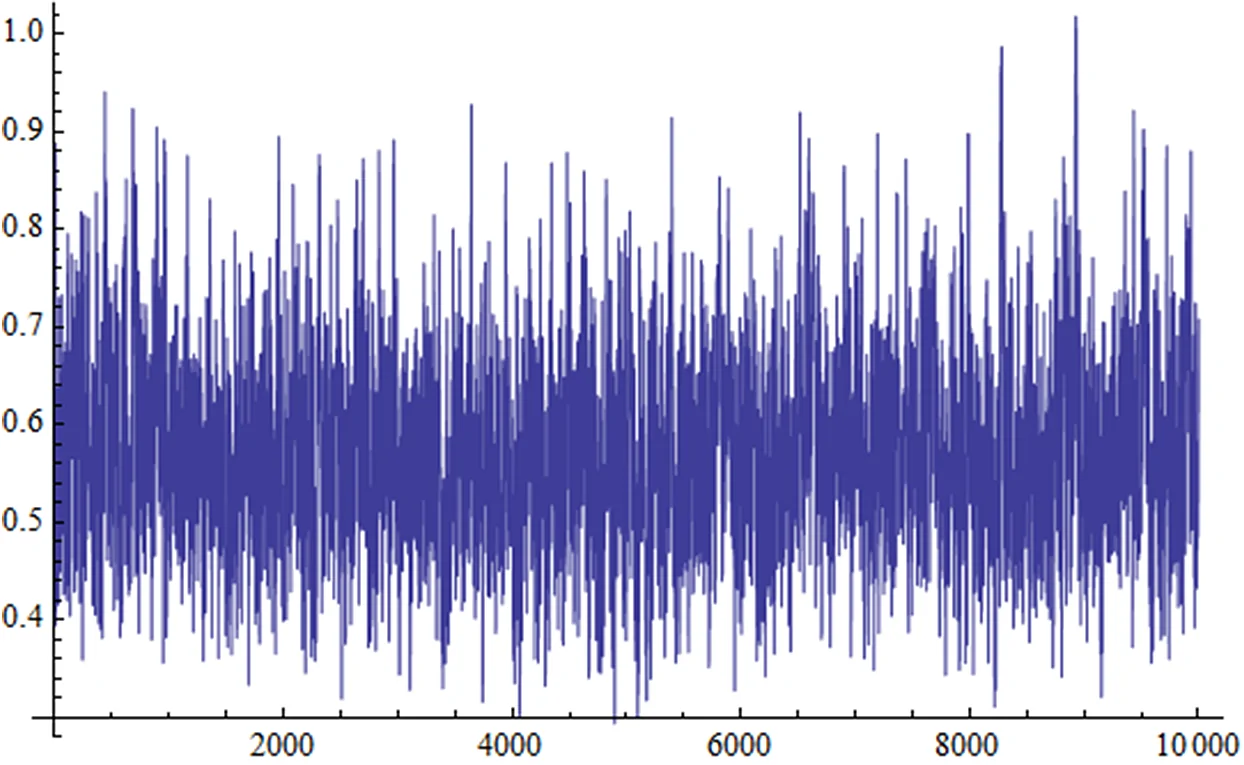
Simulation number of the parameter *b* generated by MCMC method.

**Fig 8:**
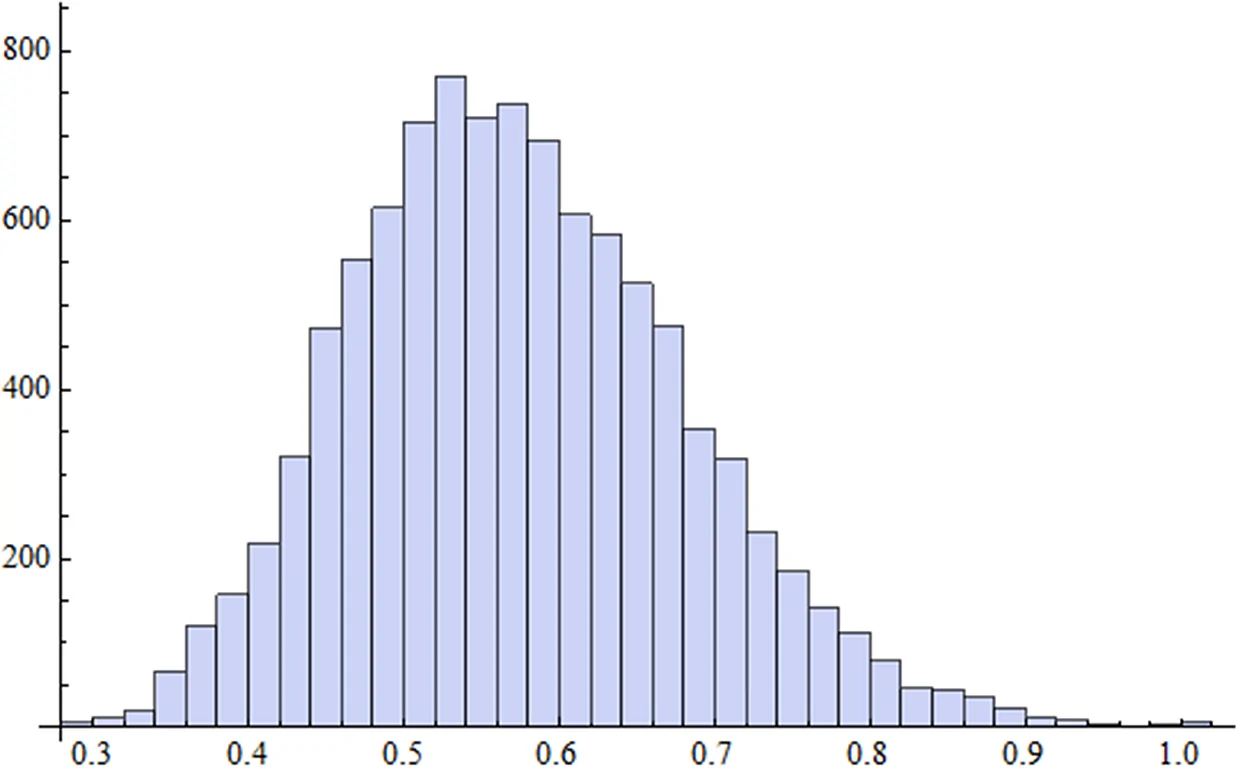
Histogram of posterior distribution of the parameter *b.*

**Fig 9:**
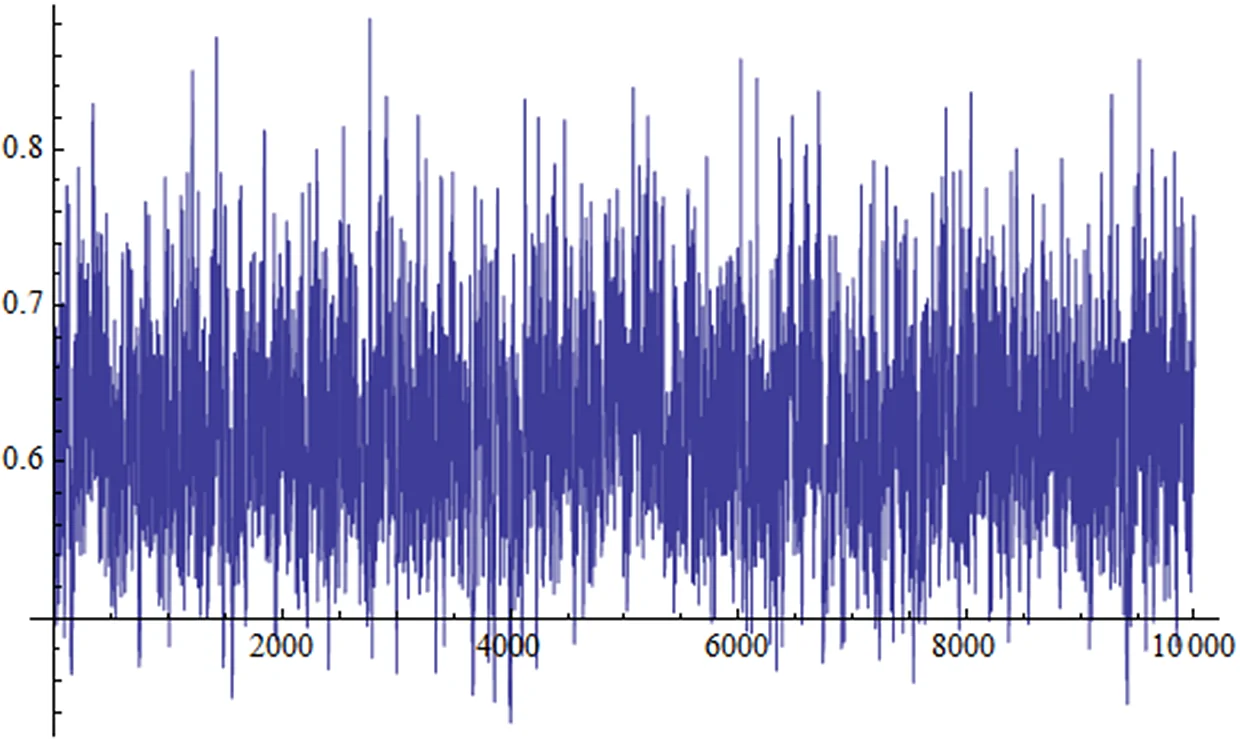
Simulation number of *CV* generated by MCMC method.

**Fig 10:**
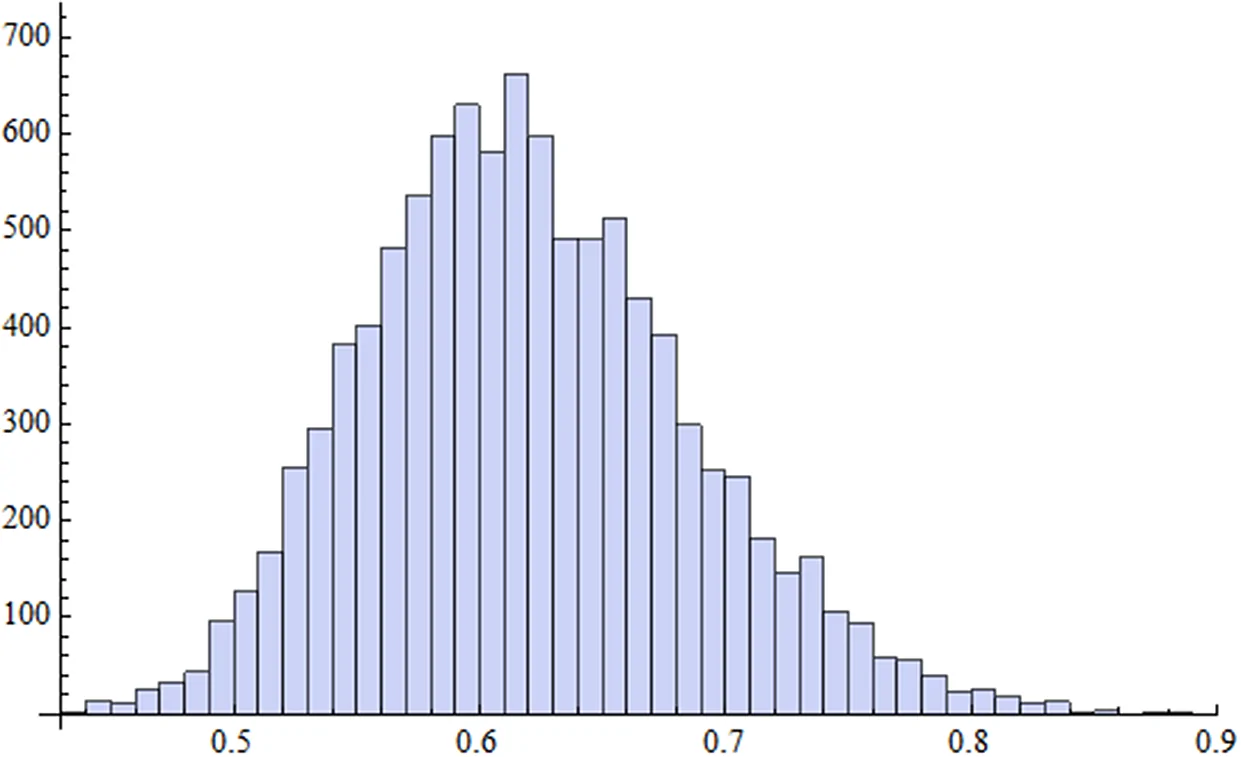
Histogram of posterior distribution of *CV.*

Figs 5–10 display the samples drawn from the posterior distribution. To assess the convergence of MCMC under Bayesian approaches, we used trace plots. These plots showed good mixing and convergence of the chains, indicating that the MCMC algorithm had reached stationarity. The trace plots also showed that a burn-in period of 1000 iterations is more suitable to ensure that the chain has converged to the target distribution.

## 5. Simulation study

To investigate the performance of the proposed estimation methods, an extensive Monte Carlo simulation study is conducted. The study considers the estimation of the CV for the WE distribution under an adaptive Type-II HPCS across a range of scenarios. The complexity of both the distribution and the censoring scheme makes theoretical comparisons analytically challenging. Hence, simulation provides an effective framework for investigating estimator accuracy and the performance of the associated confidence and credible intervals. The evaluation is based on several key performance metrics, including absolute biases (ABs), mean squared errors (MSEs), coverage probabilities (CPs), and average lengths (ALs) of the 95% bootstrap confidence intervals and Bayesian credible intervals for the CV.

The simulated data were generated using the Algorithm 3, as reported in Ng et al. [11], considering two parameter settings of the WE distribution: (*a*,*b*)=(1.5,0.5) and (0.5,1.5). To examine the impact of censoring, we applied various combinations of the CS parameters *m*, *n*, *r* and η, as outlined in Tables 2–5. The specific CSs used in this study are defined as follows

**Table 2:** ABs and MSEs of estimates of CV when *a* = 1.5 and *b* = 0.5.

η	*n*	*m*	Sampling scheme	ML		Bayes P^0^		Bayes P^1^	
				ABs	MSEs	ABs	MSEs	ABs	MSEs
0.5	30	15	(14^0^,15)	0.0573	0.154	0.0423	0.142	0.0273	0.095
			(15^1^)	0.0363	0.124	0.0283	0.118	0.0013	0.089
			(15,14^0^)	0.0003	0.111	0.0057	0.107	0.0267	0.085
	30	20	(19^0^,10)	0.0033	0.098	0.0167	0.091	0.0297	0.079
			(5^0^,10^1^,5^0^)	0.0307	0.084	0.0097	0.079	0.0187	0.071
			(10,19^0^)	0.0187	0.075	0.0177	0.072	0.0157	0.066
	30	30	(30^0^)	0.0097	0.069	0.0097	0.067	0.0107	0.058
	50	30	(29^0^,20)	0.0167	0.059	0.0157	0.054	0.0013	0.047
			(1,1,0,...,1,1,0)	0.0087	0.051	0.0027	0.045	0.0087	0.039
			(20,29^0^)	0.0157	0.035	0.0077	0.032	0.0047	0.028
	50	50	(50^0^)	0.0297	0.031	0.0007	0.028	0.0117	0.021
1.5	30	15	(14^0^,15)	0.0153	0.115	0.0133	0.112	0.0193	0.095
			(15^1^)	0.0137	0.103	0.0113	0.100	0.0263	0.092
			(15,14 ^0^)	0.0037	0.098	0.0097	0.095	0.0087	0.088
	30	20	(19 ^0^,10)	0.0063	0.087	0.0007	0.082	0.0157	0.078
			(5^0^,10^1^,5^0^)	0.0007	0.081	0.0097	0.075	0.0107	0.071
			(10,19 ^0^)	0.0157	0.075	0.0177	0.071	0.0003	0.065
	30	30	(30 ^0^)	0.0097	0.069	0.0097	0.065	0.0067	0.058
	50	30	(29^0^,20)	0.0077	0.056	0.0003	0.051	0.0217	0.045
			(1,1,0,...,1,1,0)	0.0257	0.051	0.0127	0.045	0.0137	0.039
			(20,29 ^0^)	0.0497	0.044	0.0187	0.039	0.0037	0.032
	50	50	(50 ^0^)	0.0137	0.038	0.0267	0.031	0.0063	0.025

Note: In the sampling scheme, $i^{j}$ indicates that constant number *j* of units randomly removed at *i* steps, *j* = 0 indicate no units are removed at *i* steps. *i* without power mean randomly removed *i* unit at one step.

**Table 3:** CPs and ALs of 95% bootstrap confidence intervals and Bayesian credible intervals for CV when *a* = 1.5 and *b* = 0.5.

η	*n*	*m*	Sampling scheme	Boot-p		Bayes P^0^		Bayes P^1^	
				CPs	ALs	CPs	ALs	CPs	ALs
1.0	30	15	(14^0^,15)	0.89	0.425	0.90	0.411	0.91	0.350
			(15^1^)	0.90	0.401	0.91	0.391	0.90	0.342
			(15,14^0^)	0.91	0.375	0.92	0.371	0.93	0.332
	30	20	(19^0^,10)	0.92	0.351	0.90	0.348	0.94	0.318
			(5^0^, 10^1^, 5^0^)	0.92	0.215	0.93	0.210	0.96	0.182
			(10,19^0^)	0.93	0.201	0.94	0.198	0.94	0.181
	30	30	(30^0^)	0.95	0.192	0.93	0.190	0.94	0.171
	50	30	(29^0^,20)	0.94	1.873	0.95	1.868	0.95	1.842
			(1,1,0,...,1,1,0)	0.93	1.815	0.93	1.807	0.97	1.792
			(20,29^0^)	0.93	1.800	0.93	1.791	0.92	1.776
	50	50	(50^0^)	0.94	1.745	0.94	1.735	0.93	1.715
2.0	30	15	(14^0^,15)	0.90	0.315	0.91	0.311	0.92	0.292
			(15^1^)	0.91	0.301	0.91	0.294	0.92	0.284
			(15,14^0^)	0.92	0.287	0.93	0.281	0.93	0.274
	30	20	(19^0^,10)	0.93	0.275	0.93	0.272	0.93	0.265
			(5^0^,10^1^,5^0^)	0.92	0.245	0.95	0.238	0.93	0.229
			(10,19^0^)	0.93	0.222	0.94	0.215	0.95	0.195
	30	30	(30^0^)	0.94	0.211	0.93	0.207	0.93	0.184
	50	30	(29^0^,20)	0.94	0.200	0.95	0.195	0.95	0.179
			(1,1,0,...,1,1,0)	0.94	0.182	0.94	0.180	0.93	0.162
			(20,29^0^)	0.90	0.171	0.94	0.164	0.94	0.151
	50	50	(50^0^)	0.94	0.162	0.93	0.158	0.95	0.135

Note: In the sampling scheme, $i^{j}$ indicates that constant number *j* of units randomly removed at *i* steps, *j* = 0 indicate no units are removed at *i* steps. *i* without power mean randomly removed *i* unit at one step.

**Table 4:** The ABs and MSEs of estimates of CV when *a* = 0.5 and *b* = 1.5.

η	*n*	*m*	Sampling scheme	ML		Bayes P^0^		Bayes P^1^	
				ABs	MSEs	ABs	MSEs	ABs	MSEs
0.5	30	15	(14^0^,15)	0.0573	0.154	0.0423	0.142	0.0273	0.095
			(15^1^)	0.0363	0.124	0.0283	0.118	0.0013	0.089
			(15,14^0^)	0.0003	0.111	0.0057	0.107	0.0267	0.085
	30	20	(19^0^,10)	0.0033	0.098	0.0167	0.091	0.0297	0.079
			(5^0^,10^1^,5^0^)	0.0307	0.084	0.0097	0.079	0.0187	0.071
			(10,19^0^)	0.0187	0.075	0.0177	0.072	0.0157	0.066
	30	30	(30^0^)	0.0097	0.069	0.0097	0.067	0.0107	0.058
	50	30	(29^0^,20)	0.0167	0.059	0.0157	0.054	0.0013	0.047
			(1,1,0,...,1,1,0)	0.0087	0.051	0.0027	0.045	0.0087	0.039
			(20,29^0^)	0.0157	0.035	0.0077	0.032	0.0047	0.028
	50	50	(50^0^)	0.0297	0.031	0.0007	0.028	0.0117	0.021
1.5	30	15	(14^0^,15)	0.0153	0.115	0.0133	0.112	0.0193	0.095
			(15^1^)	0.0137	0.103	0.0113	0.100	0.0263	0.092
			(15,14 ^0^)	0.0037	0.098	0.0097	0.095	0.0087	0.088
	30	20	(19 ^0^,10)	0.0063	0.087	0.0007	0.082	0.0157	0.078
			(5^0^,10^1^,5^0^)	0.0007	0.081	0.0097	0.075	0.0107	0.071
			(10,19 ^0^)	0.0157	0.075	0.0177	0.071	0.0003	0.065
	30	30	(30 ^0^)	0.0097	0.069	0.0097	0.065	0.0067	0.058
	50	30	(29^0^,20)	0.0077	0.056	0.0003	0.051	0.0217	0.045
			(1,1,0,...,1,1,0)	0.0257	0.051	0.0127	0.045	0.0137	0.039
			(20,29 ^0^)	0.0497	0.044	0.0187	0.039	0.0037	0.032
	50	50	(50 ^0^)	0.0137	0.038	0.0267	0.031	0.0063	0.025

Note: In the sampling scheme, $i^{j}$ indicates that constant number *j* of units randomly removed at *i* steps, *j* = 0 indicate no units are removed at *i* steps. *i* without power mean randomly removed *i* unit at one step.

**Table 5:** ALs and CPs of 95% bootstrap confidence intervals and Bayesian credible intervals for CV when *a* = 0.5 and *b* = 1.5.

η	*n*	*m*	Sampling scheme	Boot-p		Bayes P^0^		Bayes P^1^	
				CPs	ALs	CPs	ALs	CPs	ALs
1.0	30	15	(14^0^,15)	0.90	0.395	0.91	0.382	0.92	0.344
			(15^1^)	0.91	0.381	0.92	0.371	0.90	0.315
			(15,14^0^)	0.90	0.375	0.92	0.365	0.93	0.302
	30	20	(19^0^,10)	0.92	0.361	0.93	0.352	0.95	0.279
			(5^0^,10^1^,5^0^)	0.94	0.349	0.93	0.341	0.93	0.265
			(10,19^0^)	0.92	0.332	0.93	0.314	0.95	0.254
	30	30	(30^0^)	0.95	0.325	0.93	0.301	0.94	0.238
	50	30	(29^0^,20)	0.93	0.311	0.92	0.291	0.96	0.225
			(1,1,0,...,1,1,0)	0.92	0.300	0.94	0.283	0.96	0.211
			(20,29^0^)	0.92	0.287	0.93	0.269	0.94	0.202
	50	50	(50^0^)	0.94	0.271	0.94	0.254	0.93	0.187
2.0	30	15	(14^0^,15)	0.91	0.346	0.90	0.333	0.94	0.315
			(15^1^)	0.91	0.325	0.92	0.320	0.93	0.301
			(15,14^0^)	0.92	0.275	0.93	0.268	0.93	0.235
	30	20	(19^0^,10)	0.92	0.264	0.92	0.260	0.93	0.222
			(5^0^,10^1^,5^0^)	0.92	0.239	0.94	0.231	0.95	0.211
			(10,19^0^)	0.92	0.224	0.92	0.211	0.94	0.200
	30	30	(30^0^)	0.94	0.209	0.93	0.202	0.94	0.189
	50	30	(29^0^,20)	0.91	0.200	0.94	0.198	0.93	0.182
			(1,1,0,...,1,1,0)	0.91	0.193	0.94	0.187	0.95	0.179
			(20,29^0^)	0.93	0.182	0.94	0.179	0.94	0.170
	50	50	(50^0^)	0.92	0.175	0.93	0.170	0.95	0.169

Note: In the sampling scheme, $i^{j}$ indicates that constant number *j* of units randomly removed at *i* steps, *j* = 0 indicate no units are removed at *i* steps. *i* without power mean randomly removed *i* unit at one step.

**Table:** 

$\begin{array}{c} \left((n-m-1{)}^{0},m\right) \end{array}$	$\begin{array}{c} r_{i}=0 \end{array}$ for all *i* < *m*, and $\begin{array}{c} r_{m}=n-m. \end{array}$
$\left(m,(n-m-1{)}^{0}\right)$	$r_{1}=n-m$ and $r_{i}=0.$ for all 1<i≤m.
(*m*^1^)	$r_{i}=1$ for all 1≤i≤m.
$\left((\frac{n-m}{2}{)}^{0},(n-m{)}^{1},(\frac{n-m}{2}{)}^{0}\right)$	$r_{i}=1$ for all $\frac{n-m}{2}<i<n-\frac{n-m}{2},r_{i}=0$ otherwise
(1,1,0,...,1,1,0)	$r_{i}=r_{i+1}=1,r_{i+2}=0$ for all $1<i<\frac{m}{3}.$

In the Bayesian framework, two types of prior distributions were examined: (i) a non-informative prior (*P*^0^), defined as a uniform distribution over [0,∞) for both *a* and *b*, which produces a posterior distribution proportional to the likelihood.; and (ii) an informative prior (*P*^1^) with hyperparameters $\left(a_{1}=5,b_{1}=5,a_{2}=5,b_{2}=0.05\right)$ when (*a*,*b*) = (0.01, 0.01), incorporating prior knowledge into the inference. Bayesian estimates and their corresponding 95% credible intervals were obtained under the squared error loss function using 11,000 MCMC samples, with the first 1,000 iterations discarded as burn-in.

All numerical computations in this study were performed using Mathematica (Version 10). Following the estimation procedures described in Section 4, ML and Bayes point estimates, together with their corresponding 95% bootstrap confidence intervals and Bayesian credible intervals, were obtained for *a*, *b*, and the CV. The estimated values were then used to compute the ABs, MSEs, CPs, and ALs associated with the 95% bootstrap confidence intervals and Bayesian credible intervals for the CV. For the construction of a bootstrap confidence interval, the bootstrap estimate of *CV* based on *N* replications, the Monte Carlo standard error decreases at a rate proportional to $\frac{1}{\surd N}$. But, increasing the number *N* of replications beyond 500 produced negligible changes in the estimated standard errors and confidence intervals, confirming that 500 replications were adequate.Thus, with *N* = 500, the simulation error is typically small enough for reliable inference in many applied settings. The simulation results, based on 500 replications of data generation, are reported in Tables 2 and 3 for (*a*,*b*) = (1.5,0.5) and in Tables 4 and 5 for (*a*,*b*) = (0.5,1.5). From these tables, we observe the following:

1. The proposed Bayesian method performs consistently well for censored samples.
2. The Bayesian CV estimates have smaller ABs and MSEs than the ML estimates, especially under informative priors.
3. The ABs and MSEs of the CV estimates decrease as *m* or the effective sample ratio *m*/*n* increases, indicating improved accuracy with more information.
4. The ALs of both bootstrap confidence intervals and Bayesian credible intervals decrease as the proportion *m*/*n* increases, reflecting greater precision in the interval estimates.
5. The intervals based on ML estimates, noninformative priors, and informative priors all achieve CPs close to the nominal 0.95 level for large samples, with intervals based on informative priors showing superior performance in small samples.
6. Bayesian credible intervals generally demonstrate better performance than bootstrap confidence intervals, resulting in shorter ALs and higher CPs, especially under informative priors.

## 6. Conclusion

This study presents statistical methods for estimating the CV of the WE distribution under an adaptive Type-II HPCS. Both ML and Bayesian approaches are employed for point and interval estimation, with Bayesian inference implemented via MCMC. A comprehensive simulation study, together with an illustrative example, highlights the effectiveness of the proposed methods, particularly under informative priors in the Bayesian framework. According to the results, the adaptive CS enhances estimation accuracy by effectively balancing test duration and information yield. Overall, the proposed estimation procedures provide a flexible and efficient framework for CV estimation in life-testing scenarios with incomplete data.

## Supporting information

S1 File See the supplementary code in S1 File. (PY)
